# Systematic Review: Comparison of the Main Variables of Interest in Publications of Canine Bite Accidents in the Written Press, Gray and Scientific Literature in Chile and Spain, between the Years 2013 and 2017

**DOI:** 10.3390/ani11030893

**Published:** 2021-03-21

**Authors:** Carmen Luz Barrios, Valentina Aguirre, Alonso Parra, Carlos Pavletic, Carlos Bustos-López, Sandra Perez, Carla Urrutia, Josefa Ramirez, Jaume Fatjó

**Affiliations:** 1 Escuela de Medicina Veterinaria, Facultad de Ciencias, Universidad Mayor, Camino La Pirámide 5750, Huechuraba 8580745, Chile; smp.vergara@gmail.com (S.P.); carla.urrutiar@mayor.cl (C.U.); josefaaramirez.19@gmail.com (J.R.); 2 Cátedra Fundación Affinity Animales y Salud, Universitat Autònoma de Barcelona, Parque de Investigación Biomédica de Barcelona, C/Dr. Aiguader 88, 08003 Barcelona, Spain; jaumefatjo@gmail.com; 3 Departamento Disciplinario de Ciencias de la Documentación, Universidad de Playa Ancha, Viña del Mar 2360072, Chile; val.aguirre.olea@gmail.com; 4 Oficina de Zoonosis y Control de Vectores, Ministerio de Salud de Chile, Monjitas 565, Oficina 1008, Santiago 8320070, Chile; alonsoparra@minsal.cl (A.P.); carlos.pavletic@minsal.cl (C.P.); 5 Departamento de Ciencias Básicas, Facultad de Ciencias, Universidad Santo Tomás, Av. Ejército Libertador 146, Santiago 8320000, Chile; cbustoslopez@santotomas.cl

**Keywords:** bites, dog bites, dog aggression, dog bites review

## Abstract

**Simple Summary:**

Dog bites are a major public health problem throughout the world. The different types of information sources that exist in relation to this issue are influencing decision making to control and prevent these incidents. For this reason, the present study aims to compare the main variables of interest in the publications of dog bite accidents in the written and grey press and scientific literature in Chile and Spain, between 2013 and 2017. The results showed that sensationalist variables in dog bite articles are reported more frequently in the press literature compared to the indexed and grey literature. Examples of these variables are involvement of potentially dangerous breeds, articles with death reports, among others. In conclusion, an improvement in the quality of the information that reaches the population about dog bites could be achieved through better and more fluid communication between scientists and journalists who publish on this topic.

**Abstract:**

Dog bites are a major public health problem, with consequences such as physical injury, psychological trauma, transmission of zoonoses, infections, and economic costs. For this reason, it is necessary to develop preventive programs, which require quality information to support the authorities’ decision-making and to raise public awareness about the application of the proposed measures. The objective of this review was to analyze the press, indexed and gray dog bite literature published during the 2013–2017 period. During that period, 385 articles from three sources of information were analyzed: Press literature, scientific literature, and gray literature. Of these, the greatest amount of information corresponding to the context and the aggressor animal was found in the press literature, where it was recorded that the greatest number of records reported in the Chilean articles were caused by potentially dangerous breeds (87.50%), having significant differences with the gray literature (*p* = 0.030), and in Spain, the greatest number of attacks was also made by potentially dangerous dogs 91.30% (21/23), statistically significant differences with the gray literature (*p* = 0.002) and with the indexed (*p* < 0.001). In the case of the scientific and gray literature, the greatest amount of information was found about the victim of the attack and the treatments applied to them. In these cases, the highest percentage of victims included in the reports contained both sexes for the two literatures (44.62% and 87.71%, respectively). Regarding the treatment applied, in the scientific literature in most of the reports, the patients received washings, rabies vaccine, and tetanus vaccine (46.26%) and presented significant differences in Chile with the information contained in the gray literature (*p* = 0.023), in Spain with the gray (*p* = 0.017) and with the press (*p* = 0.023). In conclusion, the press literature differs in multiple variables with the information reported in the scientific literature and, in some cases, with the gray literature. The reason why the material that is being distributed to the population would not coincide in multiple relevant variables in other literature and the representative reality of the problem is the basis for this topic.

## 1. Introduction

Accidents due to animal bites are an important national and international public health problem, both due to their frequency and their health care requirements, affecting children and adults regardless of socioeconomic status [1]. The dog is the animal most frequently involved in this type of incident [2,3,4,5].

Every year, more than seven million people in the world are bitten by dogs. In the United States, for example, there is an average of 337,103 annual visits to health facilities for this cause [6], which ranks 10th in nonfatal involuntary accidents in children aged one to four years [7].

As a result of canine bites, important consequences arise, among which are physical injuries, psychological trauma, transmission of zoonoses [8], infections after bites [9,10], and economic costs, both for the state and the victim, who may require medical attention and reconstructive procedures [11]. The consequences derived from bites vary according to factors related to the characteristics of the aggressor animal and the affected person, generating injuries of varying severity [1] that impact the physical [12,13] and psychological [14] integrity of the victim. In addition, the risk of zoonosis transmission associated with these incidents is added [15,16,17]. In extreme cases, the death of the patient may occur [18,19,20]. There are different risk factors for fatal dog bites, which can be classified according to their relationship with the characteristics of the victim, the biting dog, and the context of the attack [21].

Given the consequences and risk factors mentioned above, it is necessary to develop programs with efficient measures to prevent bite incidents [4]. However, to carry out these programs, it is necessary to have good information to support the decision-making of the authorities, and that makes the population responsive to applying the proposed measures.

One of the bibliographic sources that aims to disseminate scientific content to the population is the press literature, which should be as accurate, complete, and balanced as possible in the publication of information for its readers [22]. Within this type of literature, articles on public health can be found, including reports of dog bites [23]. Despite the contribution that this information can make to the community, there is controversy about the real contribution of this type of literature and whether it achieves the objective of informing society in an adequate manner or whether it has the opposite effect [23].

Currently, there are multiple scientific articles focusing on the analysis and, in some cases, comparison of different sources of information from the medical literature (the type of literature where dog bite cases should be published) [24,25,26,27]. Previous studies have suggested that the information provided by the mass media has produced more harm than contributions to public health [28]. This may be due, among other things, to publications with deficient or inadequate information about the risks, benefits, and costs of medical treatments [29,30], as well as a lack of disclosure of conflicts of interest, which are very rarely declared in this type of literature [31,32], unlike scientific literature that is obliged to declare them. This problem could also be present in the specific literature on canine bites.

Not having information with a good level of scientific or medical quality (accuracy, completeness, or balance) may lead to an error in the population’s decision-making on the central topic of discussion, in this case, dog bites. This is why, by publishing quality news related to health, people will be able to make better decisions in this area [26]. This can only be achieved by safeguarding the completeness of the publications, thus minimizing distortions [33]. In addition, the information provided must have an adequate critical tone that considers limitations, risks, and challenges, among others, avoiding highlighting only a particular part of the information [34,35]. Unfortunately, previous research shows that the quality of this type of literature is low [26,30,36].

The purpose of this research was to compare the reporting frequency of variables of interest associated with incidents of canine bites in different data sources (scientific literature, gray, and written press), easily accessible for the population of Chile and Spain. These countries have similar cultural roots but have differences in the level of socioeconomic development and different influences from neighboring countries. Further, a law on Potentially Dangerous Dogs exists in Spain, unlike Chile, which lacked this legislation at the time of this study.

## 2. Materials and Methods

### 2.1. Search Strategy

This research corresponds to a systematic review of scientific literature, gray literature, and written press articles from Chile and Spain between 2013 and 2017.

The information was obtained according to the following criteria: primary (publication that publishes original research results) and secondary articles on canine bite incidents published between 2013 and 2017 (literature that synthesizes the information available, e.g., systematic reviews, epidemiological studies, and studies on injuries caused by canine bites). The following scientific literature databases were used: Scopus, PubMed, Science Direct, and Web of Science Core Collection. In addition, studies registered in gray literature databases were compiled, such as Safety Lit and Lilacs, and news from the written press published in El País, El Mundo, and La Vanguardia (Spain) and El Mercurio, Las Últimas Noticias, and La Tercera (Chile). These are the three main newspapers in Spain and Chile in terms of coverage and number of readers, respectively.

For the selection of articles by categories, the following criteria were considered for each of the types of literature analyzed:

(a) Scientific (indexed) literature: All articles that came from indexed scientific journals were included in the scientific databases mentioned above.

(b) Press literature: All press articles on the central issues that originated in some of the three Chilean or three Spanish newspapers mentioned in this section during the period of this study.

(c) Gray literature: All articles from the previously mentioned gray literature information bases focused on the central themes of this study. Within these, all thesis publications, popular science magazines, discussion articles related to canine bites, which come from scientific organizations, were considered.

Articles in Spanish and English were considered.

Multiple keywords were used in the collection of articles (Table 1). Each word was searched in both English and Spanish, with only a few exceptions in one language (e.g., canin *) in response to the first search strategy test, as it sometimes only has responses in one language. In addition, the truncation (*) was used to extend the search to all words with a common beginning (e.g., canin *). Likewise, we use different Boolean Operators (Table 2).

### 2.2. Inclusion Criteria

Only references published between 2013 and 2017 were considered, corresponding to primary and secondary articles: original research articles, reviews, case reports, conference abstracts, and guidelines, among others.

### 2.3. Exclusion Criteria

**Figure 1:**
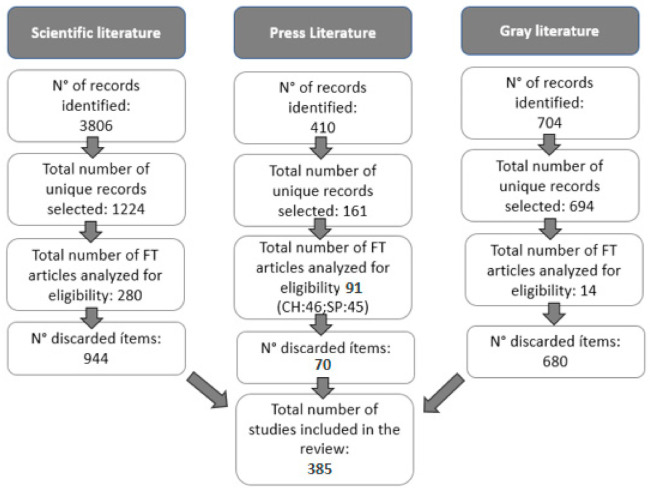
Flow chart used for the systematic review. Meaning of abbreviations: CH: Chile, SP: Spain, FT: Full text and N°: Number.

Publications of the editorial type, comments, letters, and book reviews, among others, were excluded. In addition, results that included the following terms NOT (insect bites OR tick bites OR snake bites OR fly bites OR sand flies) were discarded. Regarding the phrase “cat bites”, it was decided not to include it due to the fact that the phrase “dog bites” was found on multiple occasions next to (NEAR) the articles retrieved during the first search mentioned above. In the initial search, 4920 records were found, corresponding to scientific, press, and gray literature (Figure 1). These articles went through four stages of filtering: identification, selection, eligibility, and inclusion. Finally, 385 articles were selected for analysis, divided into 280 scientific articles, 91 press articles (45 Spain; 46 Chile), and 14 gray literature articles.

### 2.4. Types of Variables Analyzed

The variables that were analyzed in the present study were distributed into four categories: information about the victim in attack incidents, information about the biting animal in canine attack incidents, information about the attack context in canine bite incidents, and characteristics of lesions and treatment. Each group of variables was described for use in the analysis and stratified with the same objective (Table 3). Previously, some general characteristics of the publications have been evaluated, such as type of information (primary or secondary) and profession of the first author of the article. The latter has been classified depending on the profession of this researcher: Doctor, Veterinarian, Public Health specialist, forensic, journalist, and other scientists, considering the latter as all researchers who do not fit into the aforementioned classifications.

### 2.5. Statistical Analysis

The analysis aimed to compare the frequency of registration of the main variables of interest in scientific, gray, and press literature. All the information collected was stored and tabulated in Microsoft Excel 2016^®^ spreadsheets. From the data obtained, we analyzed the differences between the frequencies of reports of the main variables of interest related to canine bite incidents recorded in the scientific, gray, and written press literature, using the proportions comparison test (Z-test), with a level of 95% confidence. For this, the statistical program Minitab^®^ v.16 was used.

## 3. Results

Three hundred and eighty-five articles were selected for analysis, divided into 280 indexed literature articles, 91 newspaper articles, and 14 gray literature articles. In these publications, we identified and analyzed the main variables of interest related to the general characteristics of the publication, characteristics of the person bitten (Table 4), characteristics of the biting animal (Table 5), context of the attack and lesions-treatment produced by the canine bites.

### 3.1. General Characteristics of the Publications

Regarding the general characteristics of the publications, 100% (91/91) of the press literature consisted of articles focused on dog bites only. This was separated into articles corresponding to the bibliographic sources of the Chilean press and another group of articles from the Spanish press. Both were 100% focused on dog bites: Chile (46/46) and Spain (45/45). In the indexed literature and gray literature, 87.14% (244/280) and 85.71% (12/14) of the publications made reference to canine bites.

Regarding the type of information, articles of primary type (Original research) were found with greater frequency in the three sources of information (press, 100% (91/91), indexed literature 97.50% (273/280), and gray literature 80% (12/14).

When analyzing the profession of the main authors, it was found that for the press literature, 100% were journalists (Chile (46/46); Spain (45/45)), for the indexed literature, the majority of the authors were doctors (84.64%) (237/280), and for the gray literature, the majority of the authors were doctors (50%) (5/10) and 50% (5/10) veterinary.

Regarding the origin of the information collected, in the press literature, 100% of the publications were interviews of press cases (Chile: 100% (46/46); Spain: 100% (45/45)), in indexed and gray literature, the largest number of records were found in previous databases, 85.36% (239/280) and 60% (6/10), respectively, where the indexed literature was statistically superior to the gray literature (*p* = 0.030).

### 3.2. Frequency of Reports with Information about the Person Bitten

For this registry, the following variables were considered: sex, age group, victim-context characteristic, and educational level (Table 4 and Table 5).

### 3.3. Frequency of Reports with Information about the Biting Animal

For this record, the following variables were considered: report of the biting dog–bitten person ownership relationship, potentially dangerous dog, knowledge of the biting dog by the affected person, and size, sex, vaccination status, and reproductive status of the dog (Table 5).

Regarding dog ownership, both in the Chilean press literature, Spanish press, indexed literature, and gray literature, they mostly had records of incidents produced by dogs that did not belong to the victim (Chilean press lit.: 75.68%, Spanish press lit.: 80.49%; indexed lit.: 51.94%; gray lit.: 75%. indicated that the dog did not belong to the victim.) In the case of the analysis of the Spanish literature for this variable, the press literature was significantly larger than the gray literature (*p* = 0.010), and the press literature was significantly larger than the indexed literature (*p* = 0.011).

**Figure 2:**
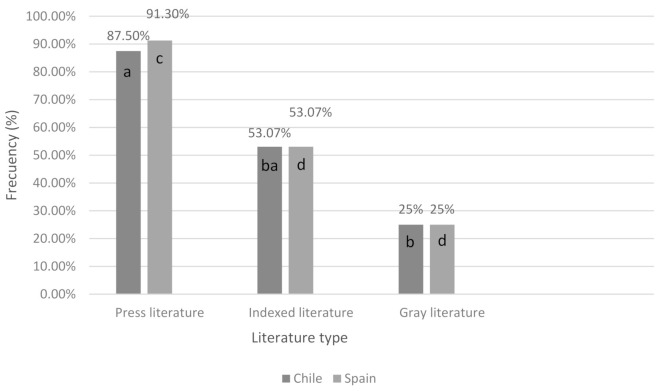
Frequency of records that report potentially dangerous dog bites in the different types of literature in Chile and Spain. The letters a and b refer to statistically significant differences in Chilean literature, and the letters c and d, statistically significant differences between Spanish literatures.

In Chile, potentially dangerous dog breeds (PDD) were reported in 87.50% (7/8) of press records, 53.07% (147/277) of indexed literature articles, and 25% (1/4) of the gray publications. There were significant differences between the press and gray literature (*p* = 0.03) (Figure 2). In the case of Spanish literature, also in the press literature, the highest percentages of reports that contained the participation of PDD breeds (91.30%) (21/23) were recorded. This was followed by indexed reading with 53 07% and finally, 25% of gray literature, had this type of record. It should be noted that a large number of articles did not contain information on the biting dog breed, 84.78% (39/46) of the Chilean press literature, 48.89% of the Spanish press articles, 82.14% (230/280) of the indexed literature, and 78.57% (11/14) of the gray literature (Figure 2).

In relation to the size of the aggressor animal, large dogs were the most reported in the press literature and indexed literature, with 91.67% (11/12) and 51.11% (138/270), respectively, and with a difference statistically significant between the two sources (*p* = 0.006).

Regarding the sex of the biting dog, this information was not included in more than 90% of the articles (100%) (46/46) press, 95% (266/280) indexed literature, and 85.71% (12/14) gray literature. There were no significant differences between any of the information sources.

The rabies vaccination status did not show significant differences between the different sources of information. Eighty percent (4/5) of newspaper articles, 65.12% (28/43) of indexed literature, and 50% (2/4) of gray literature did not refer to this variable.

Finally, 91.11% (41/45) of the press articles, 87.86% (246/280) of the indexed literature, and 92.86% (13/14) of the gray literature did not have information about the reproductive status of the biting dog.

### 3.4. Frequency of Reports with Information about the Attack Context in Bite Incidents

For this record, the following variables related to the context of the attack were considered: location of the attack, context, season of the year, and type of person–dog approach.

Regarding the location of the attack, both the Chilean (CH) and Spanish (SP) press literature had the largest number of articles with this information (CH: 82.61% (38/46) SP: 86.67% (39/45)) in comparison with the indexed literature: 15.36% (43/280), with a statistically significant difference between both literatures (*p* < 0.001). Of the multiple places of occurrence of the attacks in the press literature, in the Chilean one, the highest number of incidents occurred inside the house 52.63% (20/38), and in the Spanish one, it was different, the highest number of records was concentrated in the street 42.11% (16/38). In the case of the indexed literature, the highest number of reports occurred within the house, 39.71% (27/68), and this coincided with the gray literature, which registered the highest number of reports in this same place, 50% (3/6). It should be noted that there were no significant differences between the locations where the attacks occurred.

The variable context of the attack concentrated in the Chilean press literature and the Spanish press literature with the largest number of articles with this information (CH: 47.83% (22/46); SP: 53.33% (24/45)). A statistically significant difference with the indexed literature in the two countries of analysis (*p* < 0.001) existed.

The largest number of articles with information on the season of the year in which the attack occurred was the press literature (CH: 82.61% (38/46); SP: 86.67% (39/45)). The literature with the lowest number of articles with this information was the indexed literature (CH and SP: 9.64% (27/280)). A statistically significant difference between press literature and scientific literature was evidenced (CH: *p* < 0.001; SP: *p* < 0.001). Regarding the differences between the different seasons and the different literatures in each country, no statistically significant differences were observed in 91.66% (11/12) of the comparisons made in each country.

One of the main variables to consider in the analysis of information on variables related to the context of the attack is the frequency of reports of deaths recorded in the articles from the different sources of literature. Thus, in the case of the comparison between the Spanish press literature with the indexed literature and the gray literature, no significant differences were evident. However, when comparing the Chilean press literature and the other two sources, a statistically significant difference was found between the press and indexed literature (*p* = 0.001).

### 3.5. Characteristics of Lesions-Treatment Produced by Canine Bites

The following variables were considered for this category: number of bites, severity, treatment, and anatomical area of the lesion.

Regarding the number of bites, in Spain, 81.25% (26/32) of the articles in the written press referred to multiple bites. In the indexed literature, most reports were of single bites, 70.56% (151/214). On the other hand, in the gray literature, the highest percentage of reports referred to single bites (100%) (1/1). In Chile, the highest number of reports for the press literature was recorded as multiple bites (83.33%) (25/30), unlike the indexed and gray literature, which had a higher number of records in single bites (70.56%) (151/214) and 100% (1/1), respectively.

In the single-bite reports belonging to the articles in the literature analyzed in Spain, statistically significant differences were observed between press and indexed literature (*p* < 0.001) and between press and gray literature (*p* = 0.050). In the case of multiple bites in this country, a statistically significant difference was recorded between press and indexed literature (*p* < 0.001). In the case of the literature analyzed in Chile, statistically significant differences were observed between press and indexed literature, for multiple bites (*p* < 0.001) and for single bites between indexed and press literature (*p* < 0.001) and between the latter and gray literature (*p* = 0.038).

**Figure 3:**
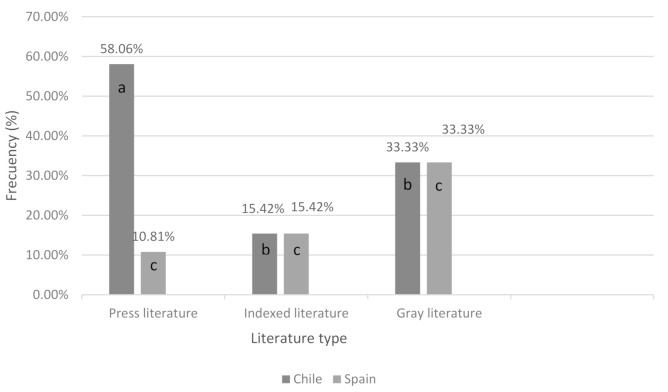
Frequency of records that report bites resulting in death in the different types of literature in Chile and Spain. The letters a and b refer to statistically significant differences in the Chilean literature, and the letter c indicates that there are no statistically significant differences between the Spanish literatures.

In relation to the severity of the injuries, in Spain, the highest number of articles referred to serious injuries, with 81.08% (30/37) in the press literature and 37.89% (86/227) in the indexed literature. In the case of the gray literature, the highest number of records was concentrated in incidents resulting in death (33.3%) (3/9). In the case of Chile, the highest number of records in the press literature (58.06%) (18/31) were in incidents resulting in death, as well as in the gray literature (33.33%) (3/9). This is in contrast to the indexed literature, which concentrated its highest number of records on serious incidents (37.89%) (86/227). In the case of articles reporting bites resulting in death, there were statistically significant differences only between the press and indexed literature pertaining to Chile (*p* < 0.001) (Figure 3).

As for the type of treatment variable, in Chile, 93.48% (43/46) of press reports and 71.43% (10/14) of gray literature records contained information on this topic. In contrast, 68.57% (192/280) of the indexed literature was based on this information. In Spain, press literature had a high number of articles without this information (77.78%) (35/45). In Chile, the most reported treatment in the press articles was surgery with 100% (3/3) of the reports. In the indexed and gray literature, the most reported treatments were washed and the application of rabies and tetanus vaccines, with 46.26% (173/374) and 100% (5/5), respectively. The indexed literature presented significant differences in Chile with the gray literature (*p* = 0.023) and in Spain with the gray (*p* = 0.017) and with the press (*p* = 0.023). In Spain, the scenario was repeated with the press literature having the largest number of records on patients treated with surgery (90%) (9/10).

In relation to the anatomical area, both in Chile and Spain, the press and indexed literature had a high percentage of articles without this information (Chile: press lit.: 52.17%, index lit.: 77.86%; Spain: press literature: 75.56%, index literature: 77.86%).

## 4. Discussion

In the results obtained in the present study, differences in multiple variables were found between the different types of literature. This is due to the different approaches to writing between the press literature and the scientific and gray literatures, as well as the different types of readers to whom the information is addressed. In the case of press literature, most of the articles written have a sensationalist bias, as this makes the news more marketable to potential readers (the general public) [26]. In contrast, in the case of scientific literature, the writing is mainly oriented towards providing an objective explanation of the problem, trying to keep bias to a minimum, and sticking to reality as closely as possible [26]. These characteristics can be seen in both indexed and gray literature. However, in the former, they are more evident due to the rigorousness of the review by editorial committees and peer review.

### 4.1. Press Literature

The greater frequency of reports with information in the press literature may be justified by the sensationalist editorial bias, which allows the general population (the target audience of these news items) to read the article [37], as well as by the way the information is collected, which deals with single episodes. Further, access to family members, witnesses, and those close to the victim, allows the press to obtain more information. This scenario is far from what happens in the indexed and gray literature, which in most cases, collect information from hospital services, with little time available to collect data, and it only comes from the clinical records of the victims. These factors limit the depth of background collection.

There is a greater tendency to generate publications with more attractive information for the reader in the written press through a greater frequency of reports where DDP participate in the incidents, both in the Spanish and Chilean literature. This may be based on the fact that this information is of great interest to readers of the press, especially of sensationalist sections, which evaluate incidents involving fatalities, serious injuries, among others [37]. In both the indexed literature and the written press, most articles did not include information on the breed of the animal involved in the incidents, which makes it difficult to classify dogs as PDD and non-PDD. However, for the articles that did incorporate this information, the majority of the print media mainly referred to PDD. In contrast, the majority of indexed and gray literature publications reported non-PDD breeds predominantly. This tendency to include PDD breeds in newspaper reports of bite accidents coincides with that reported by [38], where newspapers in the city of Calgary considered the inclusion of PDD breeds, mainly Pit bulls, to be highly relevant, contrasting with officers’ reports of dog bites recorded in the same city during similar study periods [39]. This is despite the fact that in that country, there are no laws prohibiting these breeds for dog bite control [38]. This biased treatment of certain dog breeds with respect to their involvement in bite incidents has been widely criticized in the associated literature, as discussed by [40], who compares the disparate handling of information related to this problem (tilting the balance towards PDD) with the disadvantages to which African Americans are subjected in the United States. In addition, another article showed a preference for reporting more bite incidents in lower-income communities than in higher-income communities [41]. In summary, the ethnoracial factors of dog owners, as well as their socioeconomic characteristics and the breed of dog, are factors that influence the writing of news articles [38]. On the other hand, this type of information can indirectly relate to various socioeconomic realities [38]. Similarly, it has been documented that this type of incident can lead to unfavorable perceptions of poorer communities [42,43].

On the other hand, the indexed and gray literature attempts to maintain as much objectivity in the data as possible, which can lead to differences in results, as evidenced in the present study. These types of biases may fuel misperceptions in the population about higher participation of PDD breeds compared to non-PDD breeds. This is despite studies supporting that there is no significant difference in the participation of PDD vs. non-PDD breeds [44].

The same factors explained above, in relation to the yellow editorial bias, may underpin the explanation as to why the press literature concentrated the highest number of articles with reports that included large or giant biting dog breeds in the analyzed Chilean literature. In the case of the Spanish literature, the same was true for two out of three of the literatures analyzed, as the press literature mainly recorded medium-sized breeds. The predominance of large and giant breed records in the print media due to the high level of injury severity could be explained by the fact that these types of incidents are more attractive to general readers, which is consistent with the bias associated with PDD breeds described in previous studies [38]. In the case of the indexed and gray literature, although a large proportion of these articles did not have this information, those that did may tend to mention large dogs because they tend to produce more severe injuries that motivate people to consult emergency services and, therefore, records containing this background can be accessed [45,46].

Furthermore, regarding injury severity, the literature with the highest number of articles containing this information was the press. It should be noted that in this type of literature source, the highest number of records pertained to severe injuries. This may be based, as with the previous variables, on the fact that in this type of literature, the severity of the injury is of great relevance, as it makes the news more marketable.

On the other hand, the ways of collecting information from the different types of literature predispose the press literature to have a greater variety and depth of some variables, either because of the interest of publication to enhance the reading of the target audience, or because of the way of approaching the episodes (individually, with greater access to the victims’ environment and the greater time available to gather background information on the attack), unlike the scientific literature, which has less opportunity to access a large number of variables, such as season in which the attack occurred, knowledge of the victim, and ownership of the biting animal.

With respect to the variable location of the attack, the press literature had the highest number of articles containing this information, both in the Chilean and Spanish literature. This could be justified by the fact that such details are interesting for newspaper readers, as they allow them to have a complete idea of the scene where the victim was at the time of the attack, and, therefore, journalists incorporate this information. In contrast, in the indexed literature, few articles included this variable. Although relevant to a complete understanding of the problem, other variables associated with the victim are often prioritized in health care databases, either due to lack of time or lack of awareness of the importance of this variable by health care personnel assisting the person who has been bitten [4].

The time of year when the attack occurred was more reported in print than in the indexed and gray literature, which may also be due to the method of data collection. The higher number of bite incidents occurring in spring and summer, reported in the print media, coincides with previous scientific studies [4,47], which indicate that in these seasons, people engage in a higher number of outdoor activities, having a higher level of exposure and interaction with animals on public roads, predisposing them to possible attacks.

In relation to the victim’s knowledge of the biting animal, different results were obtained in the three types of literature for the unknown animal variable, finding a clear predominance of these records in the written press. This could be explained by differences in the origin and form of data collection with which the previous variable coincides.

In the case of biting animal ownership, in Spain, most of the articles containing this information belonged to the press literature as opposed to the scientific and gray literature, which, in most of the articles, lacked this background information. The same was true for the comparison between the different types of Chilean literature. These high percentages of reports in the press literature may be justified on the grounds of access to information mentioned in the previous paragraphs. This information can contextualize the reader about the relationship the victim had with the offending animal. In the case of indexed and gray literature, the origin of the information used by these bibliographic sources is usually databases taken previously and in contexts that lack the time to compile it, so this information is omitted. It is important to note that, although this background information may be very useful for understanding the problem, it is not the priority for solving health problems in the emergency department. With respect to observed statistically significant differences on this point, in Chile, it only occurred in indexed and press literature. In the case of Spain, this difference was evident between indexed and press literature, and between gray and press literature.

It is for all the above reasons, and some others, that in the last decade, it has been suggested that publications in the popular media may be detrimental to public health [28]. It can be noted how easy it is to disseminate these data to the general population; however, newspapers do not achieve a complete understanding of health problems [29]. This hypothesis is in agreement with what is suggested by [26], who states that print media information focused on the health area has inaccuracies and biases in the analyzed news. This can be justified by deadlines, the search for an eye-catching headline, and corporate editorial pressures. This scenario is exactly what happens with biting incidents, as it is a news item that, despite being mainly found in the police sections of newspapers, must be in the appendices of medical reports. Previous studies have explained that writing with a bias to overestimate some variables could contribute to serious problems in the population when making decisions about the problems discussed in the articles [48,49]. Therefore, by reporting a higher number of incidents with high-severity injuries produced by certain dog breeds, the reader might be induced to focus attention on PDD attacks with severe consequences or death. Previous studies comparing journalistic reports with indexed literature have suggested that one of the factors that may predispose to this type of tendency could be the limited expertise of journalists, who do not have a network of expert scientific contacts to advise them and are more likely to refer to organizations related to the topic, which may have some preferences that guide the outcome of the final publication according to their own interests [50. This point is important, as previous research has suggested that news integrity is related to the number of information sources consulted, which may be associated with the universe of contacts the journalist has [26]. Another interesting point of discussion is the lack of interest that journalists of this type of news may have in educating the population about this type of problems as they do not always care about informing readers to make decisions with the best argument in the reality of the central issue [25]. This coincides with the approach previously stated by [50], who said that less experienced journalists and those working in private reporting institutions preferred to priorities entertainment in their reporting over other variables, which could lead to less comprehensive writing.

### 4.2. Indexed Literature

As explained at the beginning of this discussion, the scientific literature aims to explain the problem through objective writing and with the minimum possible bias [26], very different from the characteristics of press literature, which is influenced by public relations in the medium that writes the news [51], the social reality of the media, editors, and journalists in charge of covering medical issues [50]. Thus, in the case of the indexed literature, the articles with the highest frequency of reports with variables that are interesting for the complete explanation of the problem, without yellowish biases, such as the press, and that are a contribution to the medical approach to the injuries. This last factor is of great relevance for the frequency of information published in this type of literature since hospital services are one of the main sources of information for scientific research. Two examples of variables that contain information of interest for the analysis of the problem from a medical point of view are the number of bites and type of treatment.

Regarding the number of bites, both in Chile and Spain, in the indexed literature, there was a greater number of incidents with single bites, which differed from what was found in the press literature, which had a greater number of published attacks with multiple injuries. This difference may be explained by the arguments mentioned in the previous paragraphs, related to the different writing objectives between the press and indexed literature. On the other hand, the percentages of articles containing this information on this variable in the indexed literature were higher than other items, based on the importance of this variable in the medical approach and prognosis.

As for the treatment variable, both in Chile and Spain, the articles in the indexed literature had the highest number of publications containing this information, with a significant difference with the press literature, which could be explained by the interest in registering this variable for professionals in hospital services in the analysis of the problem.

### 4.3. Gray Literature

This type of literature did not present important differences for the understanding of the present analysis; however, it is important to highlight that it showed greater similarity in its behavior with the indexed literature than with the press literature.

## 5. Conclusions

The editorial objectives of the different sources of information may induce the inclusion of different antecedents in each of the variables of relevance to the problem analyzed, as well as in the specific weight given to each variable to be communicated. The different sources of the data used can determine relevant differences in the published results, and the lack of information on some variables can lead to an erroneous analysis of the problem. This can be reflected in the results obtained in the present study by the press and scientific literature in variables of relevance for the analysis of this problem, such as the number of articles with serious incidents or deaths, the number of bites in the incident, the approach of the dog to the victim, the location of the attack, and the participation of potentially dangerous dogs in the attacks.

An improvement in the quality of this type of article could be achieved through a better and more fluid connection between scientists dedicated to the subject of canine bites and related subjects, such as ethologists, epidemiologists, anthrozoologists, doctors, and professionals in the field of health, among others. It is important to insist on the need for human and animal health professionals to make efforts to communicate the results of their studies to the general public. It is clear that this is not the only factor responsible for the success of a publication with respect to its veracity and objectivity, but it is undoubtedly one of the fundamental pillars to improve the completeness of the publication. It should be noted that despite all the biases mentioned in the news of the written press, this type of publication keeps society’s attention on these issues. However, care must be taken not to blur the focus of the news to correctly guide the community in how to handle this important public health problem. Likewise, it is important to highlight that, although indexed and gray literature handle the information in a slightly more objective way, there is still much to be done regarding the analysis of this problem, such as having a greater number of variables of importance and ensuring that the databases used to carry out research have the largest amount of information per variable. This could perhaps be achieved by making the health personnel aware of the importance of filling in these items or obtaining data from other sources.

## Figures and Tables

**Table 1:** Keywords in English and Spanish. Multiple keywords were used in the collection of articles (Table 1). Each word was searched in both English and Spanish, with only a few exceptions in one language (e.g., canin *) in response to the first search strategy test, as it sometimes only has responses in one language.

Keywords in English	Keywords in Spanish
Dog Bites Bitten Biting “Bites and Stings” [MeSH] Epidemi * Public health Injur * Wounds Attack	Perro Canin * Mordedura Mordida (only in Press) “Mordeduras y Picaduras” [DeCS] Epidemi * Salud pública Lesiones

**Table 2:** Search strategies used in systematic review. The truncation (*) was used to extend the search to all words with a common beginning (e.g., canin *).

Search Strategy	AND	AND	AND	NOT (*)
dog bites OR “mordeduras de perro”	injuries OR wounds OR lesions OR attack	epidemiology OR epidemic OR epidemiología OR public health	bites OR biting OR bitten OR “Bites and Stings” [Mesh]	insect bites OR tick bites OR snake bites OR fly bites OR sand flies

In addition, the following inclusion and exclusion criteria were defined.

**Table 3:** Classification of the variables of interest for further analysis.

Variable	Variable Description	Variable Classification
Bitten person		
Sex	Sex of the bitten person.	Man Woman Both Not reported
Age group	Age group of the victim, measured in years	Group 1 (0–4 years) Group 2 (>4–9 years) Group 3 (>9–14 years) Group 4 (>14–25 years) Group 5 (>25–35 years) Group 6 (>35–49 years) Group 7 (>49–64 years) Group 8 (≥65 years) Not reported
Victim-context characteristics	Reported characteristics of the victim associated with the context of the attack	Sexual abuser Illegal Pit Bull breeder Housewife Student Guard Thief Military/police Unemployed Dependent worker Independent worker Tourist Not reported
Educational level	Educational category reached by the victim	No formal education Preschool Basic school Middle school Higher education Postgraduate Not reported
Biting animal information		
Report of the biting dog-victim ownership relationship	Status of ownership of the animal with a responsible person, guardian, or owner	It belonged to the victim It did not belong to the victim Not reported
Potentially dangerous dog (PDD)	Dog belonging to a breed, or its crosses, with potential aggressive characteristics in accordance with the regulations of each country or territory	Reports with PDD breed Reports with no PDD breed Not reported
Knowledge of the biting animal by the victim	Statement of knowledge of owner, address, or habitual location of the biting dog	Known Animal Unknown Animal Not reported
Biting dog size	Subjective statement of the affected person in relation to the size or height of the biting animal. The sizes of the biting animals were classified as follows: Small (less than 14 kg), medium (between 14–25 kg), large (25–50 kg), and giant (over 50 kg).	Small Medium Big Giant Not reported
Biting dog vaccination status	Declaration of validity of rabies vaccination of the biting animal	Vaccinated Not vaccinated Not reported
Biting dog sex	Sex of the dog causing the bites	Male Female Both Not reported
Reproductive status of the biting animal	Statement of reproductive status of the aggressor dog	Sterilized Not sterilized Not reported
Information about the attack context		
Location of the attack	Place where the bite incident occurred	Inside the doghouse Public space Street Park Other Not reported
Context	Situation or interaction between the affected person and the biting animal, in which the biting incident occurred related to the activity that the aggressor animal was performing at the time of the attack	Sleeping Eating Playing Fighting with another dog Person walking or running Other Not reported
Season of the year of the attack	Time of year the bite incident occurred. In the case of articles based on information from countries in the northern hemisphere, the following dates were considered: Spring: 21 March to 20 June. Summer: 21 June to 20 September. Autumn: 21 September to 20 December. Winter: 21 December to 20 March. In the case of articles from the southern hemisphere Spring: 21 September to 20 December. Summer: 21 December to 20 March. Autumn: 21 March to 20 June. Winter: 21 June to 20 September.	Spring Summer Autumn Winter Not reported
Type of approach	Circumstance in which the incident occurred regarding the approach of the person and the animal	Human to dog Dog to human Not reported
Characteristics of the lesions-treatment		
Number of bites	N°. of reported bites. The classification of single or multiple bites was considered to be the number of bites per victim.	Single Multiple Both Not reported
Severity of injury	Level of damage caused by the bite	Mild (scratch) Intermediate (tissue penetration) Severe (tissue tear) Death Not reported
Treatment type	Clinical, pharmacological, surgical, or other interventions applied to the bitten person	Wash, rabies vaccine, tetanus vaccine Antibiotic Surgery Amputation No treatment Not reported
Anatomical area of the injury	Place in the body where the injury caused by the biting animal was located.	Head and neck Upper extremity Lower extremity Other single zone Multiple zones Not reported

**Table 4:** Frequency of records that contain information about the person bitten in incidents of canine attacks in the Chilean written press, indexed and gray literature.

	Press Literature (P)		Indexed Literature (I)		Gray Literature (G)		*p*-Value		
	(n)	(%)	(n)	(%)	(n)	(%)	(G vs. P)	(P vs. I)	(I vs. G)
**Victim’s Sex Reports**	**N=**	**39**	**N=**	**251**	**N=**	**7**			
Man	29	74.4%	93	37.05%	0	0.00%	0.000	0.000	0.044
Woman	10	25.6%	46	18.33%	1	14.29%	0.517	0.282	0.785
Both	0	0.0%	112	44.62%	6	85.71%	0.000	0.000	0.031
	**N=**	**46**	**N=**	**280**	**N=**	**14**	**(G vs. P)**	**(P vs. I)**	**(I vs. G)**
Reported	39	84.8%	251	89.64%	7	50.00%	0.007	0.330	0.000
Not reported	7	15.2%	29	10.36%	7	50.00%	0.007	0.330	0.000
**Victim’s age group**	**N=**	**26**	**N=**	**356**	**N=**	**17**	**(G vs. P)**	**(P vs. I)**	**(I vs. G)**
Group 1 (0–4 years)	3	11.54%	66	18.54%	0	0.00%	0.146	0.370	0.050
Group 2 (>4–9 years)	1	3.85%	36	10.11%	1	5.88%	0.757	0.297	0.569
Group 3 (>9–14 years)	1	3.85%	27	7.58%	5	29.41%	0.018	0.480	0.002
Group 4 (>14–25 years)	6	23.08%	32	8.99%	4	23.53%	0.973	0.021	0.047
Group 5 (>25–35 years)	1	3.85%	25	7.02%	1	5.88%	0.757	0.535	0.857
Group 6 (>35–49 years)	2	7.69%	54	15.17%	1	5.88%	0.820	0.298	0.291
Group 7 (>49–64 years)	2	7.69%	58	16.29%	2	11.76%	0.000	0.245	0.620
Group 8 (≥65 years)	10	38.46%	58	16.29%	3	17.65%	0.146	0.004	0.883
	**N=**	**46**	**N=**	**280**	**N=**	**14**	**(G vs. P)**	**(P vs. I)**	**(I vs. G)**
Reported	25	54.35%	226	80.71%	4	28.57%	0.091	0.000	0.000
Not reported	21	45.65%	54	19.29%	10	71.43%	0.091	0.000	0.000
**Context-related characteristics of the victim**	**N=**	**13**	**N=**	**113**	**N=**	**3**	**(G vs. P)**	**(P vs. I)**	**(I vs. G)**
Sexual abuser	1	7.69%	1	0.88%	0	0.00%	0.620	0.063	0.870
Illegal Pit Bull breeder	0	0.00%	0	0.00%	0	0.00%	-------	------	------
Housewife	0	0.00%	16	14.16%	0	0.00%	-------	0.146	0.483
Student	2	15.38%	18	15.93%	0	0.00%	0.468	0.959	0.452
Guard	1	7.69%	2	1.77%	0	0.00%	0.620	0.185	0.816
Thief	1	7.69%	0	0.00%	0	0.00%	0.620	0.003	-------
Military/police	2	15.38%	4	3.54%	0	0.00%	0.000	0.058	0.740
Unemployed	0	0.00%	7	6.19%	0	0.00%	------	0.356	0.657
Dependent worker	3	23.08%	31	27.43%	3	100.00%	0.013	0.738	0.006
Independent worker	2	15.38%	30	26.55%	0	0.00%	0.468	0.381	0.300
Tourist	0	0.00%	4	3.54%	0	0.00%	------	0.491	0.740
Animal	1	7.69%	0	0.00%	0	0.00%	0.620	0.003	------
	**N=**	**46**	**N=**	**280**	**N=**	**14**	**(G vs. P)**	**(P vs. I)**	**(I vs. G)**
Reported	13	28.26%	40	14.29%	1	7.14%	0.102	0.017	0.452
Not reported	33	71.74%	240	85.71%	13	92.86%	0.102	0.017	0.452
**Educational level**	**N=**	**2**	**N=**	**28**	**N=**	**0**	**(G vs. P)**	**(P vs. I)**	**(I vs. G)**
No formal education	0	0.00%	0	0%	0	--------	--------	--------	--------
Preschool	0	0.00%	2	7.14%	0	--------	--------	0.696	--------
Basic school	1	50.00%	8	28.57%	0	--------	--------	0.523	--------
Middle school	0	0.00%	9	32.14%	0	--------	--------	0.338	--------
Higher education	1	50.00%	5	17.86%	0	--------	--------	0.272	--------
Postgraduate	0	0.00%	4	14.29%	0	--------	--------	0.566	--------
	**N=**	**46**	**N=**	**280**	**N=**	**14**	**(G vs. P)**	**(P vs. I)**	**(I vs. G)**
Reported	2	4.35%	9	3.21%	0	0.00%	0.427	0.693	0.496
Not reported	44	95.65%	271	96.79%	14	100.00%	0.427	0.693	0.496

P = Press literature, I = Indexed literature, G = Gray literature.

**Table 5:** Frequency of records that contain information about the person bitten in incidents of canine attacks in the Spanish written press, indexed and gray literature.

	Press Literature (P)		Indexed Literature (I)		Gray Literature (G)		*p*-Value		
	(n)	(%)	(n)	(%)	(n)	(%)	(G vs. P)	(P vs. I)	(I vs. G)
**Victim’s Sex Reports**	**N=**	**41**	**N=**	**251**	**N=**	**7**			
Man	20	48.78%	93	37%	0	0.00%	0.016	0.153	0.044
Woman	21	51.22%	46	18%	1	14.29%	0.070	0.000	0.785
Both	0	0.00%	112	45%	6	85.71%	0.000	0.000	0.031
	**N=**	**45**	**N=**	**280**	**N=**	**14**			
Reported	41	91.11%	251	90%	7	50.00%	0.001	0.762	0.000
Not reported	4	8.89%	29	10%	7	50.00%	0.001	0.762	0.000
**Victim’s age group**	**N=**	**42**	**N=**	**356**	**N=**	**17**			
Group 1 (0–4 years)	8	19.05%	66	19%	0	0.00%	0.053	0.936	0.050
Group 2 (>4–9 years)	6	14.29%	36	10%	1	5.88%	0.366	0.405	0.569
Group 3 (>9–14 years)	2	4.76%	27	8%	5	29.41%	0.008	0.506	0.002
Group 4 (>14–25 years)	7	16.67%	32	9%	4	23.53%	0.540	0.113	0.047
Group 5 (>25–35 years)	2	4.76%	25	7%	1	5.88%	0.859	0.582	0.857
Group 6 (>35–49 years)	7	16.67%	54	15%	1	5.88%	0.273	0.799	0.291
Group 7 (>49–64 years)	4	9.52%	58	16%	2	11.76%	0.000	0.253	0.620
Group 8 (≥65 years)	6	14.29%	58	16%	3	17.65%	0.745	0.738	0.883
	**N=**	**45**	**N=**	**280**	**N=**	**14**			
Reported	34	75.56%	226	81%	4	28.57%	0.001	0.422	0.000
Not reported	11	24.44%	54	19%	10	71.43%	0.001	0.422	0.000
**Context-related characteristics of the victim**	**N=**	**5**	**N=**	**113**	**N=**	**3**			
Sexual abuser	1	20.00%	1	1%	0	0.00%	0.408	0.001	0.870
Illegal Pit Bull breeder	1	20.00%	0	0%	0	0.00%	0.408	0.000	-----
Housewife	0	0.00%	16	14%	0	0.00%	-----	0.365	0.483
Student	0	0.00%	18	16%	0	0.00%	-----	0.332	0.452
Guard	0	0.00%	2	2%	0	0.00%	-----	0.764	0.816
Thief	0	0.00%	0	0%	0	0.00%	-----	-----	-----
Military/police	2	40.00%	4	4%	0	0.00%	0.000	0.000	0.740
Unemployed	0	0.00%	7	6%	0	0.00%	-----	0.566	0.657
Dependent worker	1	20.00%	31	27%	3	100.00%	0.028	0.714	0.006
Independent worker	0	0.00%	30	27%	0	0.00%	-----	0.182	0.300
Tourist	0	0.00%	4	4%	0	0.00%	-----	0.669	0.740
Animal	0	0.00%	0	0%	0	0.00%	-----	-----	-----
	**N=**	**45**	**N=**	**280**	**N=**	**14**			
Reported	5	11.11%	40	14%	1	7.14%	0.668	0.567	0.452
Not reported	40	88.89%	240	86%	13	92.86%	0.668	0.567	0.452
**Educational level**	**N=**	**0**	**N=**	**28**	**N=**	**0**			
No formal education	0	-----	0	0%	0	-----	-----	-----	-----
Preschool	0	-----	2	7%	0	-----	-----	-----	-----
Basic school	0	-----	8	29%	0	-----	-----	-----	-----
Middle school	0	-----	9	32%	0	-----	-----	-----	-----
Higher education	0	-----	5	18%	0	-----	-----	-----	-----
Postgraduate	0	-----	4	14%	0	-----	-----	-----	-----
	**N=**	**45**	**N=**	**280**	**N=**	**14**			
Reported	0	0.00%	9	0.032	0	0.00%	-----	0.223	0.496
Not reported	45	100.00%	271	0.968	14	100.00%	-----	0.223	0.496

P = Press literature, I = Indexed literature, G = Gray literature.

## Data Availability

Not applicable.
